# Prevalence of Hysterectomy among Gynecological Surgeries in a Tertiary Care Hospital

**DOI:** 10.31729/jnma.5315

**Published:** 2020-12-31

**Authors:** Tara Manandhar, Sarita Sitaula, Baburam Dixit Thapa, Ajay Agrawal, Achala Thakur

**Affiliations:** 1 Department of Obstetrics and Gynecology, B. P. Koirala Institute of Health Sciences, Dharan, Nepal

**Keywords:** *hysterectomy*, *laparoscopy*, *leiomyoma*, *pelvic organ prolapse*

## Abstract

**Introduction:**

Hysterectomy is the most common gynecological procedure. Over the last decade, the minimally invasive approach has been practiced more frequently. Fibroid uterus being the most common indication for hysterectomy justifies this minimal approach, however, whenever feasible, vaginal hysterectomy can be the preferred route. The objective of this study was to find out the prevalence and indication of hysterectomy among major gynecological surgeries in a tertiary care hospital.

**Methods:**

A descriptive cross-sectional study was done at a tertiary care hospital among 1912 patients who had major gynecological surgeries from January 2017 to December 2019. Ethical clearance was obtained from the institutional review committee (ref. no. ACD 935/076/077). Convenient sampling was used. Statistical analysis was done using Statistical Package for Social Sciences version 21.0. Point estimate at 95% Confidence Interval was calculated along with frequency and proportion for binary data.

**Results:**

During the study period, there were 1,912 major gynecological surgeries and the prevalence of hysterectomy was 1,131 (59.15%) (56.94-61.35 at 95% Confidence Interval). Fibroid uterus was the most common clinical indication for hysterectomy which was done in 397 (35.10%) patients, followed by uterovaginal prolapse in 254 (22.46) patients, adnexal mass in 210 (18.56%), and abnormal uterine bleeding in 117 (10.34%) patients.

**Conclusions:**

Hysterectomy, being the most common gynecological surgery, selection of the most appropriate route is of paramount importance. As for any other surgery, it is not without complication and hysterectomy should always be justified. With the advancement in the conservative approaches, these organ-preserving options should be explored rigorously before opting for hysterectomy.

## INTRODUCTION

Hysterectomy is the most common surgery in Gynecology. Depending on nature of disease and patient's characteristics, it is performed via abdominal, vaginal or minimally invasive approach.^1-4^ the fourth National Family Health Survey (NFHS-4 Rate of a hysterectomy varies between the countries ranging from 2.13-3.62/1000 in Germany to 5.4/1000 in United States.^5^ Fibroid uterus, abnormal uterine bleeding (AUB), pelvic organ prolapse and benign ovarian tumours are common indications for hysterectomy.^1,2,4-8^

Abdominal hysterectomy is still the most commonly used approach. But, there have been preferences for vaginal and laparoscopic approaches recently.^4–6,9–12.^

The uterus is an organ of self-being for women, so hysterectomy apart from its defined complications inherits great dissatisfaction for women. As per recommended by expert panel, 70% of hysterectomy were not appropriate.^12^ There have been improvements in the organ-preserving options especially for benign indication and these options should be explored.^5,13^

This study aimed to find out the prevalence and indication of hysterectomy among gynaecological surgeries in a tertiary care center.

## METHODS

A descriptive cross-sectional study was done at B.P Koirala Institute of Health Sciences, Dharan, Nepal, from January 2017 to December 2019. Ethical clearance was obtained from the institutional review committee (IRC Ref. No. ACD 935/076/077) before starting the study. All the patients who had undergone hysterectomy at BPKIHS during the study period were included in the study. Hysterectomy done outside and referred to BPKIHS for various other reasons, post-partum hysterectomy and emergency hysterectomy were excluded from the study. The convenient sampling technique was used. The sample size was calculated using the formula:

$$\begin{array}{ll} \text{n} & \text{= }{\text{Z}}^{\text{2}}\times \text{p}\times \text{(1}-\text{p)}/{\text{e}}^{\text{2}}\\ & \text{= }{\left(1.96\right)}^{\text{2}}\times (0.5)\times (1-0.5)/{(\text{0.03)}}^{\text{2}}\\ & \text{= }1067.11\\ & =1067 \end{array}$$

Where,

n = required sample size
Z = 1.96 at 95% Confidence Interval (CI)
p = population proportion, 50%
e = margin of error, 3%

Taking a 10% non-response rate, the sample size became 1174. However, 1912 patients were enrolled in the study.

The case records of all the patients were reviewed and the patients' demography, indications of surgery, surgical approach, complication, and mortality were noted. Abdominal hysterectomy included total abdominal hysterectomy (TAH), TAH with bilateral salpingectomy, bilateral salphing-oophorectomy (BSO), and radical hysterectomy, but these subgroups were analyzed separately. Apart from radical hysterectomy which was analyzed separately, the hysterectomy done as a part of staging laparotomy for malignancy was included in TAH. Minimal invasive hysterectomy included Laparoscopic-assisted vaginal hysterectomy (LAVH) and total laparoscopic hysterectomy (TLH). All the Histopathological diagnosis was noted and was compared with the preoperative diagnosis to see the accuracy and justify the need for hysterectomy. We used the Statistical Package for Social Sciences version 21.0 and point estimate at 95% Confidence interval was calculated along with frequency and proportion for binary data.

## RESULTS

During the study period, there was a total of 1912 major gynecological surgeries, out of which 1131 (59.15%) (56.94-61.35 at 95% CI) patients underwent a hysterectomy. Among 1131 hysterectomized patients, 1110 (98.14%) patients underwent open and the rest 21 (1.85%) had minimally invasive surgery. Abdominal hysterectomy was performed in 855 (75.59%) patients and vaginal hysterectomy in 255 (22.54%) patients. Minimal invasive surgery was done in 21 (1.85%) patients. Out of 21 cases, 2 (9.52%) Total Laparoscopic Hysterectomy required conversion to open. The type of surgical approach had remained constant in the last three years except for transabdominal hysterectomy (TAH) with bilateral salpingectomy which was high in the year 2018 (Table 1).

**Table 1:** Types of Hysterectomy.

Type of Hysterectomy Open	Year			Frequency n (%)
	2017	2018	2019	
Radical hysterectomy	4	2	3	9 (0.79)
TAH^*^	13	1	1	15 (1.32)
TAH + bilateral salphingectomy	25	85	42	152 (13.43)
TAH + BSO^†^	211	243	225	679 (60.03)
VH + PFR	83	86	86	255 (22.54)
Minimal Invasive				
TLH^‡^	7	6	5	18 (1.59)
LAVH^§^	1	1	1	3 (0.26)
Total	344	424	363	1131 (100)

* TAH: total abdominal hysterectomy,

† BSO: bilateral salphing-oophorectomy,

‡ TLH: total laparoscopic hysterectomy,

§ LAVH: Laparoscopic-assisted vaginal hysterectomy.

Fibroid uterus was the most common clinical indication for hysterectomy which was done in 397 (35.10%) patients, followed by uterovaginal prolapse in 254 (22.46) patients, adnexal mass in 210 (18.56%), and abnormal uterine bleeding in 117 (10.34%) patients. These four were the major 978 (86.47%) indications for hysterectomy. There was a significant decrease in the cases of Adnexal mass and Pre-invasive disease in the year 2019, but the overall common disease pattern remains the same over the last three years (Table 2).

**Table 2:** Indications for Hysterectomy.

Indication	Year			Frequency n (%)
	2017	2018	2019	
Abdominopelvic Mass	13	13	23	49 (4.33)
Adnexal Mass	71	87	52	210 (18.56)
Adenomyosis	10	12	11	33 (2.92)
Abnormal uterine bleeding with Failed Medical Management	30	40	47	117 (10.34)
Endometrial Polyp	0	0	1	1 (0.09)
Pyometra	2	1	1	4 (0.35)
Fibroid				
Cervical Fibroid	1	0	0	1 (0.09)
Fibroid Uterus	114	155	128	397 (35.10)
Uterovaginal Prolapse	82	86	86	254 (22.46)
Pre-invasive Disease	10	19	4	33 (2.92)
Malignancy	11	11	10	32 (2.83)
Total	344	424	363	1131 (100)

Complications were seen among 40 (3.53%) patients who underwent major gynecological surgeries. Twenty-one patients had an iatrogenic injury during surgery: bowel injury in 15 (1.3%), bladder in 5 (0.4%), and 1 (0.09%) had a major vascular injury. In the 15 patients who had bowel injury, 13 patients underwent primary repair and had an uneventful recovery. One patient had a sigmoid injury requiring colostomy and in another patient, ileal perforation was detected on the 2nd postoperative day, requiring laparotomy and ileostomy. Both of these patients had successful restoration of the bowel continuity. All the bladder injuries were detected intraoperatively and repaired with an uneventful outcome. Fourteen patients (1.24%) had surgical site infection and three (0.27%) patients had a postoperative chest infection. We had two (0.17%) mortality. One patient who had a ruptured dermoid cyst developed postoperative septicemia and another one operated on for carcinoma ovary died due to chest infection (Table 3).

**Table 3:** Intraoperative and postoperative complications.

Variables	Frequency n (%)
Intraoperative	
Bowel injury	15 (1.32)
Bladder injury	5 (0.44)
Major vascular injury	1 (0.09)
Operative time (mean±SD)	81.5±37.51
Postoperative	
Hospital stay (median days)	3
Wound infection	14 (1.24)
Chest infection	3 (0.27)
Mortality	2 (0.17)

Similarly, the histopathological examination of the specimen was done to confirm the diagnosis. The histopathological analysis of the specimens revealed leiomyoma 450 (39.79%) as the most common finding. There were 189 (16.73%) cases of the benign tubo-ovarian disease in which mature cystic teratoma 91 (48.15%) was the commonest finding. Fifty-one (4.5%) patients had malignancy and ovarian carcinoma 22 (43.1%) was the commonest malignant disease. Mature cystic teratoma was the most common cause for both abdominopelvic mass 12 (24.49%) and adnexal mass 76 (36.19%). Yearly analysis of histopathological diagnosis revealed the drop in the cases of Endometriosis and pre-invasive disease in the year 2019. Table 4 shows the histopathological diagnosis of the specimen in the study duration from 2017 to 2019 (Table 4).

**Table 4:** Histopathological diagnosis of the specimen.

Histopathological Diagnosis	Year			Frequency n (%)
	2017	2018	2019	
Adenomyosis	28	33	37	98 (8.66)
Endometriosis	11	26	5	42 (3.71)
Endometritis	3	1	6	10 (0.88)
Endometrial Polyp	2	1	3	6 (0.53)
Leiomyoma	129	178	143	450 (39.79)
Lipoma Uterus	1	0	0	1 (0.09)
Pyometra	0	0	1	1 (0.09)
Chronic Cervicitis	82	84	87	253 (22.37)
Tubo-ovarian disease (benign)	63	62	64	189 (16.71)
Mature Cystic Teratoma	31	30	30	91 (48.15)
Serous Cystadenoma	13	9	7	29 (15.34)
Hemorrhagic Cyst	9	6	13	28 (14.81)
Mucinous Cystadenoma	4	4	3	11 (5.82)
Paratubal Cyst	3	5	3	11 (5.82)
Others	3	8	8	19 (10.05)
Pre-invasive disease	8	14	1	23 (2.03)
Borderline	1	3	3	7 (0.62)
Malignancy	16	22	13	51 (4.51)
Uterus	2	3	6	11 (21.57)
Ovary	8	11	3	22 (43.14)
High Grade Serous Carcinoma	6	7	1	14
Others	2	4	2	8
Cervix	5	7	4	16 (31.37)
Squamous Cell Carcinoma	5	6	3	14
Others	0	1	1	2
Metastatic Adenocarcinoma	1	1	0	2 (3.92)
Total	344	424	363	1131

The mean age of the patients undergoing hysterectomy was 49.35±10.30 years (range 26 to 86 years) and 471 (41.64%) hysterectomy was done in the age group between 41-50 years. (Table 5)

**Table 5:** Demographic profile of the patients undergoing hysterectomy.

Variables	Frequency n (%)
Age Group	
≤ 30	8 (0.70)
31 - 40	230 (20.33)
41 - 50	471 (41.64)
51 - 60	248 (21.92)
61 - 70	128 (11.31)
71 - 80	40 (3.53)
81 - 90	6 (0.53)
Parity	
Nulligravida	64 (5.65)
P1-P5	939 (83.02)
>P5	138 (12.20)
Comorbid conditions	
Hypertension	121 (10.69)
Diabetes	54 (4.77)
Hypothyroidism	43 (3.80)
Respiratory disease	23 (2.03)
Others	21 (1.85)

## DISCUSSION

Hysterectomy was the most common (59.15%) gynecological surgery performed in our institute. A total of 62.68% hysterectomies were performed in the patients aged less than 51 years, which is the common age for hysterectomy as reported by Morgan et al, where they had 78.3% (n=302,547) hysterectomy in patient age less than 55 years.^1^ There seems to be a decreasing trend in hysterectomy in the reproductive age group women in the USA, and also an overall decline in the rate of hysterectomy (36.4% decline from 2002 to 2010), but we had no significant change in the rate of hysterectomy in last three years and was highest in the year 2018 (37.4 %).^1,14^

The most common indication for hysterectomy was the fibroid uterus followed by pelvic organ prolapse, adnexal mass, and AUB. These four were the major indication for hysterectomy (86.47%) in our study. Fibroid uterus remains the most common indication for elective hysterectomy worldwide, constituting 39.9 to 73.7% of all hysterectomy.^3–5^ The incidence of pelvic organ prolapse is decreasing in developed countries, but it is still the second most common indication and constitute a major bulk for hysterectomy (16.3%-18.83%) in developing countries.^1,4,14,15^ This trend was also similar in our study (pelvic organ prolapse =22.46%), whereas Toma et al. from Canada had DUB (26.4%) as the most common indication for hysterectomy followed by leiomyoma uterus (16%).^7^

Over the past decade, there has been a steadily increasing use of minimally invasive gynecological surgery: 3-15.5% in 2002-2006 to 36-43.4% in 2012-2013.^1,10,14^ We have seen progress in laparoscopic surgery in our institute as well. From 2002 to 2018, there was 4 fold increase in laparoscopic procedure in our institute, however, total cases of hysterectomies were low (TLH= 7.7%, LAVH=3%).^16^ In the last three years, we had 21 cases of minimally invasive hysterectomy (1.8% of total hysterectomy), similar to the other part of the developing countries.^4,15^ This low rate may be explained by the low resources, the cost factor, and expertise available, in the government hospital of rural areas. If we can overcome this hurdle, there is a great place for laparoscopic hysterectomy, as seen in the study by Karki et al, where he reported 1012 cases of TLH in two years, from a private institute in eastern Nepal.^17^ This disparity was also seen in the study from Finland (n=10,110) where the rate of Laparoscopic hysterectomy was high in a private hospital (LH=67%) compared to the local hospital (LH 19%).^6^ Abdominal hysterectomy remains the common route ranging from 47.8% to 83.6%.^10,18,19^ Abdominal hysterectomy was also the commonest route in our study (75.59%), and the rate was higher as compared with others because of the low incidence of minimally invasive hysterectomy.^1,18,20^ However, this rate of abdominal hysterectomy was comparable to the studies (70.2% - 83.6%) where the laparoscopic approach was not or minimally used.^3,4,7,19,21^ Vaginal hysterectomy is the most recommended and preferred route whenever feasible, with the least morbidity and better outcomes.^11,13^ VH is mostly performed for pelvic organ prolapse and was true in our study as well where we performed VH exclusively for pelvic organ prolapse only (99.6%).^3,4^ With the proper patient selection, Kovac et al performed VH in 98.9% (10,975 out of 11,094) patients with benign pathology, suggesting that abdominal and laparoscopic route might have been overused or misused.^9,10^

In our study, the total complication rate was 3.53% which was lower than the study from Finland (n=10,110), where their complication rate was 17.1% - 23.3% but they had a significant decline in overall complication rate over 10 years (17.5% in 1996 to 14.7% in 2006).^6,10^ Whereas in the study from India, the overall complication rate was 8.5%, with a significantly higher complication rate in the abdominal approach (10.9% vs 2.1% in vaginal approach).^4^ Injury to urinary tract was most commonly seen during hysterectomy than in other major gynecological surgery and had been reported in 0.2 to 1.1% cases and was higher in laparoscopic hysterectomy, whereas the bowel injury has been reported to range from 0.1 to 1%.6,^10,18^ The rate of organ injury in our study was comparable to these study groups. In the 15 patients who had bowel injury, 14 were detected intraoperatively. All the bladder injuries were detected intraoperatively and repaired with an uneventful outcome, similar to the study by Makinen et al where they detected 60-70% of organ injuries intraoperatively.^10^ 0.1% had external iliac artery injury requiring massive blood transfusion. This rate of vascular injury is also low in our study compared to the reported incidence of 2.1-3.1%.^6^

The reported incidence of surgical site infection after hysterectomy ranges from 0% to as high as 22.6%.^18^ We had 1.24% patients who developed surgical site infection: superficial SSI (9), deep SSI (4), and organ-specific (1). Three patients (0.26%) developed a postoperative chest infection. This rate of chest infection is comparable to other studies (0-2.16%).^18^ In the study by Makinen et al, out of 10,110 hysterectomies, they had a mortality rate of 0.02%, 0.04%, and 0.06% in abdominal, laparoscopic, and vaginal hysterectomy respectively. Surprisingly, their mortality rate was comparatively high for vaginal hysterectomy, which is supposed to have a low rate of morbidity.^6^ We had 0.17% mortality. One patient who had a ruptured dermoid cyst developed sepsis postoperatively and collapsed on the 7th postoperative day. Another patient who was operated on for carcinoma ovary was expired on the 10^th^ postoperative day because of postoperative chest infection.

The uterus is an organ of self-being for a female, so hysterectomy besides its procedure-related complications is a cause for dissatisfaction. In the study from the US regarding the appropriateness of hysterectomy, they found that at high as 70% of cases didn't meet the expert panel's criteria for the need for hysterectomy.^12^ We believe that hysterectomy was justified in 91.39% of our cases, after correlating with histopathological diagnosis, similar to other studies (84-91.37%).^4,15^ In 2.47% patients, we could have avoided hysterectomy as the specimen examination didn't reveal any significant pathology. Alternative treatment options like myomectomy, fibroid embolization, endometrial ablation, hysteroscopy with D&C, polypectomy, or uterine artery embolization should always be considered depending on the disease and facility available, to avoid the inappropriate hysterectomy.^5,22^

## CONCLUSIONS

Hysterectomy, being the most common gynecological surgery, selection of the most appropriate route is of paramount importance. As for any other surgery, it is not without complication and hysterectomy should always be justified. With the advancement in the conservative approaches, these organ-preserving options should be explored rigorously before opting for hysterectomy, keeping it as the last resort surgery.
